# Efficient Blended Models for Analysis and Detection of Neuropathic Pain from EEG Signals Using Machine Learning

**DOI:** 10.3390/bioengineering13010067

**Published:** 2026-01-07

**Authors:** Sunil Kumar Prabhakar, Keun-Tae Kim, Dong-Ok Won

**Affiliations:** 1 Department of Artificial Intelligence Convergence, College of Information Science, Hallym University, Chuncheon 24252, Republic of Korea; sunilprabhakar22@gmail.com (S.K.P.); ktkim@hallym.ac.kr (K.-T.K.); 2 Department of Neurology, College of Medicine, Hallym University, Chuncheon 24252, Republic of Korea; 3 Department of Population and Quantitative Health Sciences, University of Massachusetts Chan Medical School, Worcester, MA 01655, USA

**Keywords:** feature extraction, feature selection, neuropathic pain detection, classification, machine learning

## Abstract

Due to the damage happening in the nervous system, neuropathic pain occurs and it affects the quality of life of the patient to a great extent. Therefore, some clinical evaluations are required to assess the diagnostic outcomes precisely. A lot of information about the activities of the brain is provided by Electroencephalography (EEG) signals and neuropathic pain can be assessed and classified with the aid of EEG and machine learning. In this work, two approaches are proposed in terms of efficient blended models for the classification of neuropathic pain through EEG signals. In the first blended model, once the features are extracted using Discrete Wavelet Transform (DWT), statistical features, and Fuzzy C-Means (FCM) clustering techniques, the features are selected using Grey Wolf Optimization (GWO), Feature Correlation Clustering Technique (FCCT), F-test, and Bayesian Optimization Algorithm (BOA) and it is classified with the help of three hybrid classification models like Spider Monkey Optimization-based Gradient Boosting Machine (SMO-GBM) classifier, hybrid deep kernel learning with Support Vector Machine (DKL-SVM) classifier, and CatBoost classifier. In the second blended model, once the features are extracted, the features are selected using Hybrid Feature Selection—Majority Voting System (HFS-MVS), Hybrid Salp Swarm Optimization—Particle Swarm Optimization (SSO-PSO), Pearson Correlation Coefficient (PCC), and Mutual Information (MI) and it is classified with the help of three hybrid classification models like Partial Least Squares (PLS) variant classification models combined with Kernel-based SVM, ensemble classification model with soft voting strategy, and Extreme Gradient Boosting (XGBoost) classifier. The proposed blended models are evaluated on a publicly available dataset and the best results are shown when the FCM features are selected with SSO-PSO feature selection technique and classified with Polynomial Kernel-based PLS-SVM Classifier, reporting a high classification accuracy of 92.68% in this work.

## 1. Introduction

When the somatosensory system becomes affected, it leads to neuropathic pain [1]. Long-term neuropathic pain is termed as chronic neuropathic pain and when the duration is more than six months, it affects the brain and spinal cord significantly, affecting the quality of life of the patient to a great extent [2]. Multiple and effective pain management techniques are not available for people affected with neuropathic pain. Understanding and diagnosing neuropathic pain is difficult as its underlying mechanism is tough to understand and interpret. Therefore, this chronic neuropathic pain has a huge clinical challenge as it leads to constant pain for patients. There is no definite biomarker to assess the neuropathic pain condition and hence diagnosing and treating it becomes much more difficult [3]. As neuroplasticity is highly unpredictable, the complexity of neuropathic pain is very tough to understand and analyze. With the help of linear techniques, the neurophysiological correlates are extracted from the EEG signals [4]. Only simple behaviour is assumed by such linear techniques and, in multiple instances, the brain does not function as a linear system, so linear techniques have severe limitations [5]. There is always a constant shifting of neuronal dynamics in the brain so that the changes cannot be easily detected by the brain in both external and internal environments.

When the neural process works in an abnormal manner, it gives rise to severe damage in the nervous system, causing chronic neuropathic pain [6]. Due to many pathological conditions, accidents or injuries, neuropathic pain occurs. Other forms of pain can be clearly distinguished from neuropathic pain based on its chronicity factor. Neural impairment is heavily contributed by chronic neuropathic pain and a lot of other disorders are sometimes accompanied by problems such as multiple sclerosis, epilepsy, diabetic retinopathy, etc. [7]. The quality of life is impacted by this chronic neuropathic pain. The standard therapies provided by the clinical drugs do not improve the symptoms for most of the cases. Apart from the medication, physiotherapy accompanied by neurostimulation is sometimes utilized to alleviate pain [8]. Only when there are continuous efforts, sincerity, and commitment can the treatment be taken fully; otherwise, it will not provide fruitful results. A healthcare professional is required to evaluate and diagnose clinical neuropathic pain to a great extent. The standard procedure involves analyzing the full medical history of the patient followed by an in-depth physical examination and other necessary tests [9]. In spite of following all these tasks carefully, it is still difficult to achieve a definite diagnosis. In this paper, two efficient blended models are proposed for the automated diagnosis and classification of chronic neuropathic pain from EEG signals. Some of the recent and important works proposed in this field are discussed as follows.

Cohen et al. discussed the mechanism of neuropathic pain and its clinical implications [10]. The medication analysis and management of neuropathic pain disorders was analyzed by Finnerup et al. [11]. The management of neuropathic pain was analyzed well by Nishikava and Nomoto [12] and the pharmacologic management of neuropathic pain was investigated well by Nudell et al. [13]. The neuropathic pain component and its relationship with the time elapsing concept was discussed by Kim et al. [14]. The brain networks with good connectivity in patients suffering from neuropathic pain were developed by Hasan et al. [15]. The EEG signals with a slow and reduced reactivity in neuropathic pain were analyzed by Boord et al. [16]. The pain severity was quantified with the help of EEG-based functional connectivity by Haghighi et al. [17]. An adaptive neuro fuzzy inference system (ANFIS) adapted Radial Basis Function (RBF) kernel SVM was used for the classification of perceptual pain by Vatankhah et al. [18]. For discriminating against neuropathic and nociceptive pain, the identification of the brief pain inventing score was performed by Erdemoglu and Koc [19]. A novel screening measure to trace the neuropathic components in patients with back pain was identified by Freynhagen et al. [20]. The pain perception was decoded well using EEG signals for a real-time reflex system by Tayeb et al. [21]. For the analysis of the severity of chronic neuropathic pain, the resting state frontal EEG biomarkers were used by Ryu et al. [22]. A systematic review about the resting state EEG biomarkers of chronic neuropathic pain was provided by Mussigmann et al. [23]. A mechanistic review about the EEG neurofeedback for the treatment of neuropathic pain in the elderly was given by Chmiel et al. [24]. A comprehensive analysis in search of a solid biomarker for chronic pain by using EEG and machine learning was performed by Rockholt et al. [25]. The acute pain signals were detected from human EEG by Sun et al. [26]. The convolutional neural networks (CNN) were used to detect pain in the scalp-based EEG by Chen et al. [27] and the subjective perception of pain was objectively quantified with EEG by Elsayed et al. [28]. Five distinct levels of pain were distinguished with the help of EEG signal features by Nezam et al. [29]. The EEG data was analyzed thoroughly for both eyes-open and eyes-closed cases for the chronic neuropathic pain conditions by Zolezzi et al. [30]. Both linear and non-linear approaches were discussed in detail for analyzing the EEG frequency bands so that the neuropathic pain could be classified easily by Zolezzi et al. [31]. The significance of clinical and electrophysiological measures was assessed for the analysis of neuropathic pain in spinal cord injury by Wydenkeller et al. and a binary classification was performed for twenty-six subjects; a classification accuracy of 84% was obtained [32]. The central neuropathic pain was predicted in spinal cord injury based on EEG classifiers by Vuckovic et al. and a binary classification was performed for forty-one subjects; a classification accuracy of 86% was obtained [33]. The Higuchi Fractal analysis of EEG signals was analyzed as a marker of central neuropathic pain in people with spinal cord injury by Anderson et al. and a binary classification was performed for twenty subjects; a classification accuracy of 80% was obtained [34]. A quantum chaos butterfly optimization based on weighted SVM was analyzed for neuropathic pain detection from EEG signals by Bobby et al. and a three-class classification was performed for twenty-eight subjects, with a classification accuracy reported of 77.72% [35]. Multiple machine learning algorithms were developed to doubt the presence of chronic pain using the EEG data by Miller et al. and a binary classification was performed for 186 subjects; a classification accuracy of 79.6% was obtained [36]. A black-white hole pattern was proposed by Tasci et al. for the detection of chronic neuropathic pain where the proposed model gave over 99% classification accuracy across all the scenarios for the thirty-six subjects and three class classification schedules [37]. For the chronic neuropathic pain analysis, deep autoencoders and hybrid Mamba classifiers were used with advanced EEG signal processing techniques where an accuracy of 99% was reported by Senturk [38]. An EEG-based brain functional network methodology was used by Adebisi et al., where under binary classification between the neuropathic pain versus the control subjects, it achieved a higher accuracy of 97% [39]. Apart from neuropathic pain analysis from EEG signals, the EEG signals also aid in the analysis of other disorders like depression [40], and, even to understand the decoding of neural mechanisms, the EEG signals are highly useful [41]. A recent study even explored the foundation model of brain dynamics with gradient positioning and spatiotemporal masking with the help of EEG signals and it was analyzed in depth by Dong et al. [42], showing the versatility and efficacy of EEG signals for various applications.

**Figure 1:**
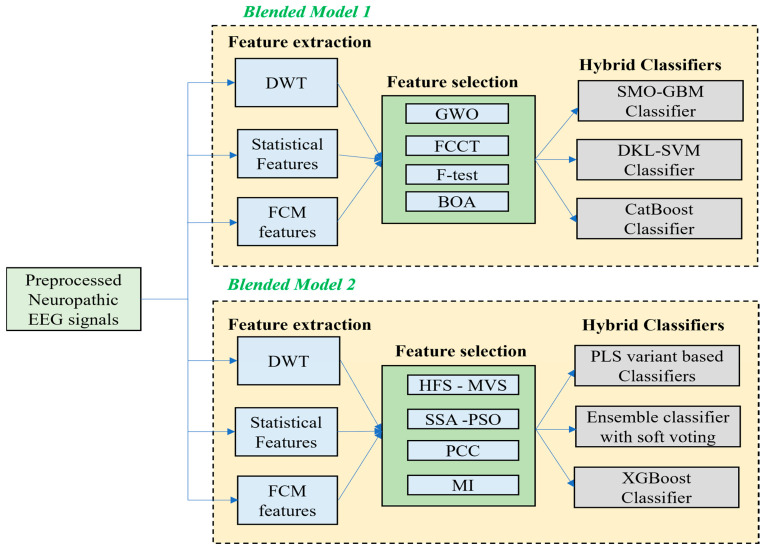
Overall Illustration of the Proposed Blended Models.

Figure 1 shows the overall representation of the proposed blended models.

The contributions of this work are summarized as follows.

(a) Once the basic pre-processing of the EEG signals is conducted using a simple Independent Component Analysis (ICA), the following two efficient blended approaches were proposed.
(b) In the first proposed efficient blended model, once the features are extracted by using DWT, statistical features, and FCM clustering techniques, the features are selected using GWO, FCCT, F-test, and BOA, and it is classified with the help of three hybrid classification models like SMO-GBM classifier, hybrid deep kernel learning with DKL-SVM classifier, and CatBoost classifier.
(c) In the second proposed efficient blended model, once the features are extracted, the features are selected using HFS-MVS technique, Hybrid SSO-PSO technique, PCC, and MI and it is classified with the help of three hybrid classification models like PLS variant classification models, ensemble classification model with soft voting strategy, and XGBoost classifier.

The organization of the paper is as follows. Section 2 discusses the various feature extraction techniques and the proposed blended model 1 is discussed in Section 3. The proposed blended model 2 is discussed in Section 4 followed by results in Section 5. Section 6 explains the discussion part in detail, followed by conclusion and future works in Section 7.

## 2. Feature Extraction Techniques

In this work, DWT features, statistical features, and FCM features are extracted thoroughly.

### 2.1. Discrete Wavelet Transform (DWT)

For the analysis of non-stationary signals, one of the most powerful time–frequency techniques is DWT [43]. It is quite easy to use and has a low computational cost, and DWT is used widely for feature extraction purposes. Using the low-pass filters and high-pass filters, the decomposition of the signal can be performed efficiently into a collection of wavelets and the representation of coefficients are performed as follows:

(1) $$F_{j,k}=\sum_{l}a_{l-2k}F_{j-1,l}$$

(2) $$G_{j,k}=\sum_{l}b_{l-2k}F_{j-1,l}$$

where the transformation level is represented by j and the number of coefficients is indicated by k. $F_{j,k}$ indicate the scaling coefficient and $G_{j,k}$ indicate the wavelet coefficient. For the scaled and wavelet functions, the coefficient of the low-pass filter and high-pass filter are specified by a and b, respectively. The width of the filter is specified by l in Equations (1) and (2). The approximate coefficient can be obtained when the removal of the high-frequency ingredients of the raw signals is accomplished successfully. The detailed coefficients can be obtained when the low frequency of the raw signals is removed by the high-pass filter. The separation of a raw signal can be accomplished successfully by shifting to a new and scaled version of a selected wavelet which is procured at any level. The sampling of the scaled coefficients and wavelet coefficients are obtained and so, the filter score length can be computed. Once the desired wavelet decomposition level is attained, the termination process can be implemented. Choosing the wavelet type is of prime importance as every wavelet has unique properties. Daubechies, Haar, Mexican, etc., are some of the commonly used wavelets and in this work (dB4) wavelet (i.e., fourth-order Daubechies is employed).

### 2.2. Statistical Features

Average Power, Standard Deviation, Kurtosis, Skewness, and Mean Absolute Value are some of the statistical features [44] computed in this work.

### 2.3. FCM Clustering Algorithm

To trace the natural patterns in a dataset, clustering can be widely used in the context of machine learning. The assigned observations in a cluster are interrelated to each other in the clustering technique. A lot of hidden data can be obtained with the help of clustering techniques in the entire dataset. The samples are divided into distinct clusters by these clustering techniques so that samples within a similar cluster are aligned closer to the samples within a dissimilar cluster. A widely used clustering algorithm is FCM and, in this algorithm, the data is prioritized to be assigned to two, three, four, or multiple clusters depending on the need of the problem [45]. Due to its practicality and easy usage, it is widely used for multiple applications in the field of pattern recognition. Most of the data is assigned to a well-prioritized cluster. Depending on the objective function minimization, the FCM is expressed as follows:

(3) $$Z=\sum_{i=1}^{N}\sum_{j=1}^{C}q_{iz}^{g}\cdot {\left\|a_{i}-C_{z}\right\|}^{2}$$

where the probability of a feature $a_{i}$ belonging to the $z^{th}$ cluster is indicated by $q_{iz}$. The $i^{th}$ feature is indicated by $a_{i}$ and the center coordinates of the $z^{th}$ cluster are assigned by $C_{z}$. The Euclidean norm is indicated by ‖ ‖ and the number of features in the database is specified by N. The number of clusters is indicated by C and the fuzziness index is indicated by g. The objective function is optimized iteratively and is used to accomplish the fuzzy partitioning. The updation of the membership $q_{iz}$ and cluster centers $C_{z}$ is indicated as follows:

(4) $$q_{iz}=\frac{1}{\sum_{k=1}^{C}{\left(\frac{\left\|a_{i}-c_{z}\right\|}{\left\|a_{i}-c_{k}\right\|}\right)}^{\frac{2}{g-1}}}$$

(5) $$C_{z}=\frac{\sum_{i=1}^{N}q_{iz}\cdot a_{i}}{\sum_{i=1}^{N}q_{iz}}$$

The data owned by multiple clusters can be reflected by the probability coefficients. A feature $a_{i}$ has a specific probability of $q_{iz}$ which belongs to a cluster z, and it is represented as follows:

(6) $$\sum_{z=1}^{C}q_{iz}=1$$

With every cluster, a prediction probability is shared and indicated by the membership functions and the distance between the class center, and the prediction probability can be assessed well.

## 3. Proposed Blended Model 1

### 3.1. Feature Selection Techniques Used in Blended Model 1

Assuming the dataset to be G which comprises S samples, each having a total of J number of features. The aim of the feature selection problem is to choose j features $\left(j<J\right)$ from the features so that the objective function $H\left(\cdot \right)$ is optimized. When the feature selection issue is solved, feature selection schemes can be encoded in a simple manner by utilizing a binary string P so that the solution of the feature selection issue can be specified well. Here, the feature selection method is indicated by P where every binary string length P equals the entire number of features $\left(J\right)$. Every feature corresponds to a particular bit in the string. If the value is one, then it implies that the feature is chosen and if the value is 0, then it implies that the feature is not chosen. Thus, a solution can be obtained by a binary string P of length J comprising ‘j’ chosen features and it is expressed in the following equation:

(7) $$P=p_{1},p_{2},\ldots ,p_{J},p_{k}\in 0,1$$

The feature selection scheme is indicated by P in the feature selection problem and $p_{k}=1$ implies that the selection of the $k^{th}$ feature is performed. For classification purposes, $H\left(\cdot \right)$ is known as the error rate. Minimizing $H\left(P\right)$ is the main intention of the feature selection where the best combination of features is found out so that the classification error is mitigated and is expressed in the following equation as follows:

(8) $$\begin{array}{cc} & \min H(P)\\ s.t. & P=\left(p_{1},p_{2},\ldots ,p_{J}\right) \end{array}$$

The above Equation (8) indicates equality constraint.

#### 3.1.1. Grey Wolf Optimization (GWO)

The social behaviour of grey wolves gave an inspiration to develop the GWO algorithm [46]. The grey wolves are modelled or rather assigned into four unique levels such as σ,ς,τ and υ. The dominant wolf in the pack is denoted by σ and it is responsible for making major decisions and assigning leadership positions. The ς wolf is secondary to σ wolf and helps in hunting activities. The τ wolf acts as a security keeper and is responsible for providing protection to the team. The rest of the wolves are categorized as υ wolves and fully follow the commands of the σ,ς,τ wolves. Therefore, the solutions of the optimization problem are specified as σ,ς,τ and υ wolves, respectively. With the guidance of the leader wolves, the prey is surrounded and hunted successfully. There is excellent cooperation among the values during hunting process so that it can track the prey efficiency and in an analogous manner, this optimization algorithm helps to find the optimal solution. The hunting procedure is categorized into three major steps.

The initial phase is called the encirclement phase. Here the prey is tracked, located, and surrounded by grey wolves usually in a pack. Depending on the approximate location of the prey, every wolf updates the position so that a siege can be formed around it successfully. When the wolf surrounds the prey, it is mathematically expressed as follows:

(9) $$\vec{P}(t+1)={\vec{P}}_{q}(t)-\vec{V}\cdot \vec{D}$$

(10) $$\vec{D}=\left|\vec{C}\vec{D}\cdot {\vec{P}}_{q}(t)-\vec{P}\left(t\right)\right|$$

where Grey Wolf position after updation is indicated by $\vec{P}(t+1)$. The corresponding distance between the prey and wolf is indicated by $\vec{D}$ and the number of iterations is indicated by t. The position vector of wolf is represented as $\vec{P}(t)$ and the position vector of prey is represented as ${\vec{P}}_{q}(t)$.

The correlation coefficient vectors are termed as $\vec{V}$ and $\vec{C}$ is computed as follows:

(11) $$\vec{V}=2\vec{v}\cdot {\vec{r}}_{1}-\vec{v}$$

(12) $$\vec{C}=2{\vec{r}}_{2}$$

The ${\vec{r}}_{1}$ and ${\vec{r}}_{2}$ indicate the random vectors in the range of [0,1]. When the number of iterations increases, the vector value $\vec{v}$ decreases in a linear manner and is computed as follows:

(13) $$\vec{v}=2-t\cdot 2/T$$

where T represents the highest number of iterations and t represents the current number of iterations.

The second phase is known as hunting phase. Once the prey location is assessed, the σ,ς,τ values precede the υ wolves successfully. The prey is captured successfully by the wolves based on mutual adjustments and cooperations. The σ,ς and τ wolves help to assess the prey’s potential position. Once it is done, their respective positions are updated using υ wolves. The leader wolf assigns a lot of information to other wolves based on the position update. Mathematically, it is expressed as follows:

(14) $$\vec{P}(t+1)=\frac{\left({\vec{P}}_{1}+{\vec{P}}_{2}+{\vec{P}}_{3}\right)}{3}$$

The vectors ${\vec{P}}_{1}$, ${\vec{P}}_{2}$, and ${\vec{P}}_{3}$ are expressed as follows:

(15) $${\vec{P}}_{1}={\vec{P}}_{\sigma }(t)-{\vec{V}}_{1}\cdot {\vec{D}}_{\sigma }$$

(16) $${\vec{P}}_{2}={\vec{P}}_{\varsigma }(t)-{\vec{V}}_{2}\cdot {\vec{D}}_{\varsigma }$$

(17) $${\vec{P}}_{3}={\vec{P}}_{\tau }(t)-{\vec{V}}_{3}\cdot {\vec{D}}_{\tau }$$

The vectors are represented as follows:

(18) $${\vec{D}}_{\sigma }=\left|{\vec{C}}_{1}\cdot {\vec{P}}_{\sigma }-\vec{P}\right|$$

(19) $${\vec{D}}_{\varsigma }=\left|{\vec{C}}_{2}\cdot {\vec{P}}_{\varsigma }-\vec{P}\right|$$

(20) $${\vec{D}}_{\tau }=\left|{\vec{C}}_{2}\cdot {\vec{P}}_{\tau }-\vec{P}\right|$$

where the distance between the search agents and σ,ς,τ wolves are indicated by ${\vec{D}}_{\sigma }$, ${\vec{D}}_{\varsigma }$, and ${\vec{D}}_{\tau }$, respectively. At a time t, the position of the σ,ς,τ wolves are indicated by the vectors ${\vec{P}}_{\sigma }(t)$, ${\vec{P}}_{\varsigma }(t)$, and ${\vec{P}}_{\tau }(t)$, respectively, and they are considered as the best solutions. Random weights are assigned by the correlation coefficients to the σ,ς,τ wolves so that the premature convergence is avoided easily. In the search space, a good diversity can be maintained with the help of these coefficients in the actual hunting process. After $\left(t+1\right)$ iterations, the search agent positions are indicated by the vector $\vec{P}(t+1)$.

**Figure 2:**
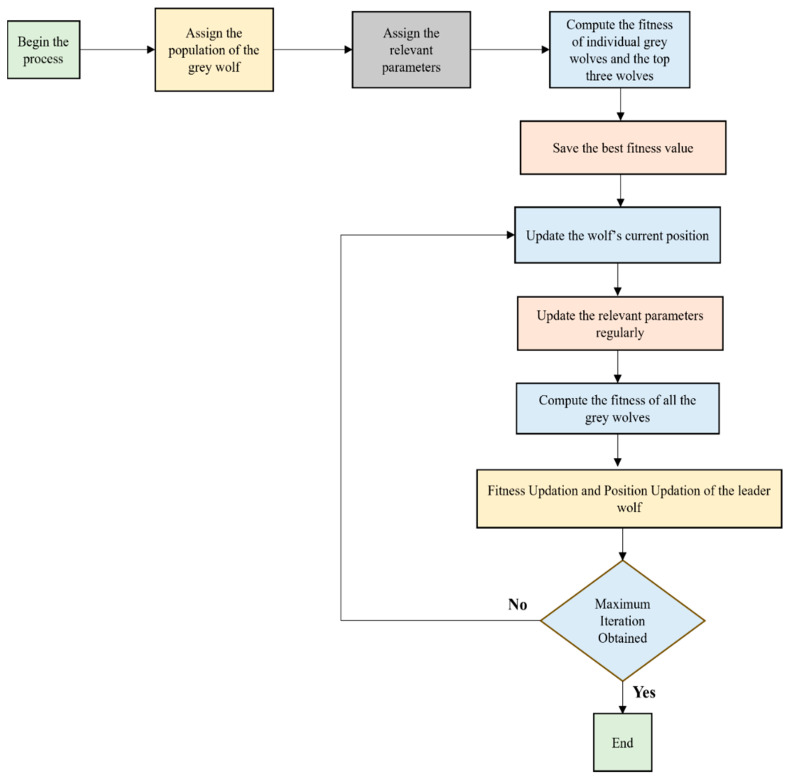
Simplified Illustration of the GWO algorithm.

The final phase is the attack-prey phase. Here the movement of the prey is stopped and the grey wolves easily charge and attack it. By means of mitigating the values of $\vec{v}$, the controlling of this process can be performed. The range of the values of the ${\vec{V}}_{1}$, ${\vec{V}}_{2}$ and ${\vec{V}}_{3}$ lies in the range of [−1, 1] in this work. If the values of the $\vec{v}$ decreases, then there is a gradual diminishing of these values as well. When $\left|V\right|<1$, the grey wolves attack the prey so that good exploitation is achieved. When $\left|V\right|>1$, the prey moves away from the grey wolves and a new prey is effectively searched in the process so that a good exploration is achieved. The simplified flowchart of the GWO is shown in Figure 2 below.

#### 3.1.2. Feature Correlation Clustering Technique (FCCT)

It is a commonly used filter technique where the collinearity problem is solved well in any dataset. When there are several features in a dataset, the model tends to become collinear, and so the ultimate performance of the model does not lie in permitting the features so that the similar data can be predicted again. The best technique is to trace the collinear features so that the number of input features can be filtered easily in the classification model. The correlation matrix is computed for every feature in the dataset utilizing any correlation technique, like Spearman’s correlation model. If a high correlation is present in between the features, then it proves that it has a lot of similar information. A hierarchical clustering is then implemented using the information obtained from the correlation data. So, a hierarchical cluster plot is developed and a specific threshold is defined so that the feature set can be targeted and extracted well [47]. From every group, a single feature is chosen so that it is made sure that it analyzes the threshold. If this threshold value is close to zero, then the generation of a greater number of clusters is prioritized and a greater number of features can be chosen. Once the feature subset is obtained, this method is stopped and the collinearity is removed easily.

#### 3.1.3. F-Test

A statistical analysis is utilized to estimate a precise maximum likelihood function so that the input features can be ranked easily by the F-test technique [48]. The significance of each feature on the output is evaluated by the F-test. F-statistics are used to quantify the importance of features. The inconsistency of the obtained features can also be quantified by the F-value. With the help of three steps, the F-test is conducted. For the individual features, the F-value is assessed. Depending on the F-values, these factors are arranged in a descending order, and then, finally, the top k-features are identified, which would possess the maximum F-value. The redundant features are eliminated by the F-test so that it is quite useful for handling multi-dimensional datasets. The overall risk of overfitting is reduced by this model to a greater extent.

#### 3.1.4. Bayesian Optimization Algorithm (BOA)

It is one of the simplest but most powerful optimization schemes which help in managing the objective functions effectively. It works in an autonomous manner and there is no need to depend on genetic operators and other conventional population-based operators. To estimate a function, a Gaussian technique is utilized so that the performance of the function is forecasted accurately. By means of considering the previously stored historical data, the efficacy of Bayesian Optimization is improved [49]. The optimal solutions can be easily searched for with the help of previously stored statistical information. When comparing both the random search and grid search algorithm, Bayesian Optimization Algorithm is highly effective when compared to even some of the newly proposed optimization algorithms.

### 3.2. Classifiers Used in Blended Model 1

The classifiers used here in the Blended Model 1 are SMO-GBM, DKL-SVM classifier, and CatBoost classifier.

#### 3.2.1. SMO-Based GBM Classifier

In this work, SMO algorithm is used with GBM classifier as follows.

**(A)**  **Spider Monkey Optimization (SMO) algorithm:**

The social activities of the spider monkeys were considered and analyzed and based on this, SMO was developed [50]. A category termed as fission-fusion was created and it was ensured that the spider monkey belonged to this category for foraging activities. The main characteristics of the fission-fusion mortals are analyzed as follows. In a population of about 40 to 50 beings, the fission-fusion category is present and can split themselves so that they can trace their food. When tracing the food particles, feminine creatures can direct the entire group. The entire group can be split into a lot of subgroups so that food can be searched efficiently if there is no sufficient food found. A solid search strategy is found by every subgroup of feminine numbers so that a good decision about the search path can be made. There is good correspondence of the individual creatures in the similar group and the neighbouring groups. To make a proper conclusion, two important parameters are required to guide the leaders, efficiently known as Local Leader (LL) limit and Global Limit (GL) limit. The H spider monkey vectors are initialized with basic vector dimensions and it is indicated as follows:

(21) $${\mathrm{SMO}}_{p}(p=1,2,\ldots ,H)$$

Assuming that there are totally ‘V’ variables in the process, every value is assigned as shown in the following equation:

(22) $${\mathrm{SMO}}_{ph}={\mathrm{SMO}}_{\min_{h}}+\mathrm{Uni}\left[0,1\right]\times \left({\mathrm{SMO}}_{\max_{h}}-{\mathrm{SMO}}_{\min_{h}}\right)$$

At $z^{th}$ local group alignment, the assignment of a new position is performed to every member. Based on the leader and the efficient knowledge of all the members in the local group, the assignment is performed as follows:

(23) $${\mathrm{SMO}}_{\begin{array}{cc} new & ph \end{array}}={\mathrm{SMO}}_{ph}+\mathrm{Uni}\left[0,1\right]\times \left({\mathrm{LL}}_{zh}-{\mathrm{SMO}}_{ph}\right)+\mathrm{Uni}\left[-1,1\right]\times \left({\mathrm{SMO}}_{th}-{\mathrm{SMO}}_{ph}\right)$$

Considering the global leader and analyzing the knowledge of all the members in the local group, the assignment of a new position is performed to every member at a global level and is represented as

(24) $${\mathrm{SMO}}_{\begin{array}{cc} new & ph \end{array}}={\mathrm{SMO}}_{ph}+\mathrm{Uni}\left[0,1\right]\times \left({\mathrm{GL}}_{h}-{\mathrm{SMO}}_{ph}\right)+\mathrm{Uni}\left[-1,1\right]\times \left({\mathrm{SMO}}_{th}-{\mathrm{SMO}}_{ph}\right)$$

The threshold “LocalLeader” limit cannot be crossed by the leader position in a local group, and so the individual portions are renewed by SMO, as per the following equation:

(25) $${\mathrm{SMO}}_{\begin{array}{cc} new & ph \end{array}}={\mathrm{SMO}}_{ph}+\mathrm{Uni}\left[0,1\right]\times \left({\mathrm{GL}}_{h}-{\mathrm{SMO}}_{ph}\right)+\mathrm{Uni}\left[0,1\right]\times \left({\mathrm{SMO}}_{ph}-{\mathrm{LL}}_{zh}\right)$$

Algorithm 1 shows the depiction of the entire process of SMO algorithm.
**Algorithm 1**: SMO Algorithm.Input Variables: LL limit, GL limit, Population, PrSteps: (a)Assess the fitness level.(b)Trace the local leader.(c)Trace the global leader.(d)While (Termination Condition is False) do(e)Assignment of novel positions ${\mathrm{SMO}}_{ph}$ is performed now(f)Implement Greedy Selection to Compute Fitness(g)Assess Fitness level as ${\mathrm{Pr}}_{p}={\mathrm{fitness}}_{p}/\sum_{p=1}^{H}{\mathrm{fitness}}_{p}$(h)Allocate the new positions to the chosen ${\mathrm{SMO}}_{ph}$ depending on ${\mathrm{Pr}}_{p}$ using Equation (22)(i)Utilize Greedy Selection to update positions of the leader of entire group and the local group leader.(j)If position updation is not performed after crossing LL limit, then ${\mathrm{SMO}}_{ph}$ is reassigned to another new group using Equation (24)(k)Investigate the global leader position for updates.(l)Subdivide the entire group if no update is found.(m)End while loop.

**(B)**  **Gradient Boosting Machine (GBM):**

An ensemble classification model comprises various simple models and is considered versatile instead of as a single classification model. The accuracy of the response parameters is improved by this GBM classification model as it tries to iteratively fit the multiple weak models [51]. At the end of every iteration, a model can be added and it utilizes a boosting centric strategy. The base classifiers are matching each other and the overall loss function is low here. The error functions are considered as squared error in this case, and though the loss function is low here, it can be chosen from a particular collection of available functions, and so a lot of flexibility is avoided by the GBM algorithm.

Assume the data provided as ${\left(p,q\right)}_{j=1}^{N}$ for the supervised learning problem. The input vector is specified as $p=\left(p_{1},\ldots ,p_{m}\right)$ and its respective label is considered as q. The loss function must be minimized, and so a function is learned by the classifier as $\hat{f}\left(p\right)=q$. The sum of the GBM algorithm is shown in Algorithm 2 as follows.
**Algorithm 2**: GBM Algorithm.Input Variables:Iteration Count $I_{n}$, loss function $\Lambda \left(q,f\right)$Base Classifier model $\eta_{N}\left(p,\gamma \right)$Input dataset ${\left(p,q\right)}_{j=1}^{N}$Procedure:(i)Allocate ${\hat{f}}_{0}$ an arbitrary value(ii)For d=1 to $I_{n}$(iii)Compute the negative gradient $n_{g}(p)$(iv)Fitting of another suitable base classifiers $\eta \left(p,\gamma_{g}\right)$(v)Compute a step size $\gamma_{g}$ for the analysis of gradient descent as follows:$\gamma_{g}=\arg\min_{g}\sum_{j=1}^{N}\Lambda \left[q_{j},{\hat{f}}_{g-1}(p_{j})+\gamma_{\eta }\left(p_{j},\gamma_{g}\right)\right]$(vi)Allocate a normal value to ${\hat{f}}_{g}$ and it is represented as follows:${\hat{f}}_{g}\leftarrow {\hat{f}}_{g-1}+\gamma_{g}\eta \left(p,\gamma_{g}\right)$(vii)End the loop.

**(C)**  **SMO Optimized GBM:**

In SMO, the solution is learned thoroughly by means of food-searching techniques by the spider monkey. Both the local best and global best values are present in the search space of the SMO solution so that the search space is utilized efficiently. The fittest survival rate must be obtained and so this SMO highly values the parameters of food-searching technique. The present positions are renewed by the spider monkeys which are heavily involved in the process of searching for food. In SMO, the experiences of the global group leader, local group leader and individual experiences are highly valued. Efficient self-organization is framed well and so the various members can be sent in different directions to search for food. Once the stagnation of the global group leaders happens, then the subdivision of that specific group happens so that the food is searched for in an efficient manner. The GBM is trained efficiently with the help of the SMO parameters as follows:

(A) The random values are initially designated.
(B) Depending on the basic optimization necessity, the robustness and versatility of every solution is examined.
(C) The global best and local best are modified accordingly.
(D) The position of the solution set is now standardized and the estimated of the stopping criteria are determined.

**Figure 3:**
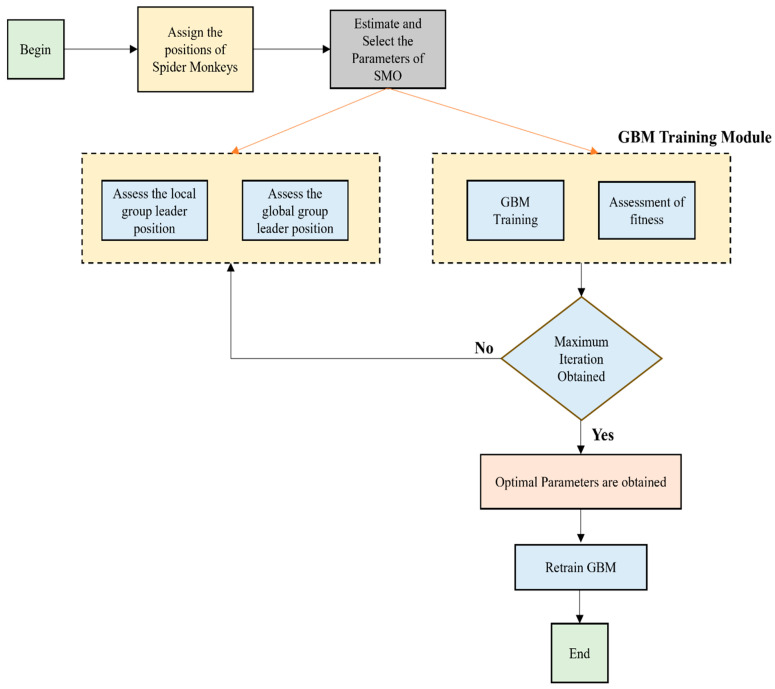
Overall Illustration of the SBO Fine-tuned GBM classifier.

The flowchart of the SBO Fine-tuned GBM classifier is shown in Figure 3 as follows.

#### 3.2.2. Hybrid Deep Kernel Learning with SVM Classifier (DKL-SVM) Classifier

SVM solves many classification problems easily. A major splitting line is utilized by SVM with two more lines termed as hyperplane [52]. Classification of an SVM can be performed into a linear or non-linear separable data. For the classification of non-linear separable data, the modification of SVM algorithm is mandatory and it is performed with the help of kernel learning. For the classification of signals, the following equations are utilized as follows:

(26) $$q_{j}\cdot s+w\geq +1\;\;for\;\;p_{j}=+1$$

(27) $$q_{j}\cdot s+w\geq -1\;\;for\;\;p_{j}=-1$$

where the ordinary space is denoted by s and the corresponding location to the coordinates of the center space is denoted by w. By means of solving the optimization problem, the splitting line is traced as follows:

(28) $$\min\frac{1}{2}{\left|\left|s\right|\right|}^{2}$$

(29) $$\mathrm{Subject}\;\mathrm{to}\;\;\;\;\;\;\;pj\left(qj\cdot s+w\right)-1\geq 0$$

The optimization problem can be solved by utilizing higher multipliers. The resolution function value is assessed easily so the determination of test data class q is managed as follows:

(30) $$f\left(q_{l}\right)=\sum_{j=1}^{n_{0}}\alpha_{j}p_{j}q_{j}q_{l}+w$$

where the support vector is denoted by $q_{j}$.

The support vector number is indicated by $n_{0}$ and the data to be classified is denoted as $q_{l}$. Multiple kernel learning [53] aims to hybridize some kernel functions into one single function as follows:

(31) $$K\left(q,q^{\prime }\right)=\sum_{n}^{N}l_{n}K_{n}\left(q,q^{\prime }\right)$$

Subject to

(32) $$l_{n}\geq 0,\sum_{n=1}^{N}l_{n}=1$$

where the number of kernel function is expressed by N. Initially, for a single kernel, an optimal hyperplane is obtained so that the optimization problem is solved as follows:

(33) $$\min\frac{1}{2}{\left\|w\right\|}^{2}+a\sum_{j=1}^{n}\zeta_{j}$$

Subject to

(34) $$p_{j}\left(q_{j}\cdot s+w\right)\geq 1-\zeta_{j}$$

The total number of misclassification data must be reduced, and so an added variable denoted as ‘a’ is incorporated and $\zeta_{j}$ denotes the penalty value.

The formulation of the optimization problem is expressed as follows:

(35) $$\min\frac{1}{2}\sum_{n=1}^{N}\frac{1}{a_{l}}{\left\|f_{n}\right\|}^{2}+a\sum_{j=1}^{n}\zeta_{j}$$

(36) $$\min L_{x}=\frac{1}{2}\sum_{n=1}^{N}\frac{1}{a_{l}}{\left\|f_{n}\right\|}^{2}+a\sum_{j=1}^{n}\zeta_{j}+\sum_{j=1}^{n}\alpha_{j}\left(1-p_{j}\sum_{n=1}^{N}f_{n}\left(q_{j}\right)-p_{j}w-\zeta_{j}\right)-\sum_{j=1}^{n}\beta_{j}\zeta_{j}$$

where the broad range multipliers are indicated by $\alpha_{j}$ and $\beta_{j}$.

#### 3.2.3. Categorical Boosting (CatBoost)

By hybridizing the words category and boosting, CatBoost is formed [54]. It has the capability to manage multiple data like text, numeric, signal, image, etc. Multiple categorical data can be overseen very well by this exceptionally proficient model and datasets of huge or limited size too can be managed by this model. A symmetric tree model is utilized by CatBoost, where similar characteristics are implemented to every tree level so that the division of the training samples can be performed into equal partitions. So, a tree can be formed which would have a depth of k and a count of $2^{k}$ leaves. In a sequential manner, the decision trees are built where the minimization loss of every tree is designed approximately. The number of trees is controlled by the initial parameters and it helps to mitigate overfitting. In case the detection of overfitting is found out, then this algorithm can stop the training early by managing the settings of the training model.

## 4. Proposed Blended Model 2

### 4.1. Feature Selection Techniques Used in Blended Model 2

The feature selection techniques used here are HFS-MVS, hybrid SSO-PSO algorithm, PCC, and MI.

#### 4.1.1. HFS-MVS Technique

When an efficient machine learning system must be built, feature selection plays quite a key role in eliminating all the noisy, redundant, irrelevant, and insignificant features. The computing time can be mitigated and the efficiency of the system can be enhanced greatly. A minimal collection of the best features can be chosen by the hybrid feature selection strategies. In an independent manner, all the techniques are utilized for feature selection and, depending on the majority voting concept, the final selection is implemented.

(A) *Correlation-based feature selection (CFS):*

The pairing of the features which are aligned together in correlation are given more importance in this technique [55]. In this technique, a low correlation value is possessed by the irrelevant features with class labels.

(B) *Least Absolute Shrinkage and Selection Operator (LASSO)*

This model penalizes the regression coefficients so that some of them are shrunk to a value of zero here [56]. The implementation of regularization process is performed here in this scheme and during the feature selection strategy, the choosing of the variables with non-zero coefficients is performed.

(C) *Local learning dependent clustering feature selection (LLCFS)*

On the clustering conceptualization idea, local learning regularization is performed so that the features can be chosen effectively [57]. In an iterative manner, the Laplacian graph is updated well.

(D) *Relief-F*

To manage multi-classification problems, the standard Relief algorithm was extended and it became Relief-F algorithm [58]. This idea is dependent on the concept of nearest neighbours and it is a supervised feature-weighted technique. In the neighbourhood of similar classes, if there is a difference in feature value, there is a decrease in the feature score. In the neighbourhood of the dissimilar classes, if there is a difference in feature value, there is an increase in the feature score.

(E) *Unsupervised Discriminative Feature Selection (UDFS):*

**Figure 4:**
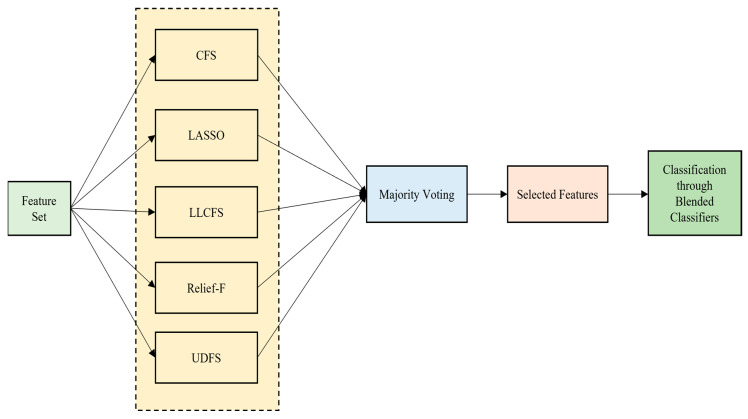
Simplified Illustration of the HFS-MVS classification model.

In this technique, the selection of features is performed in batch mode. The implementation of a versatile framework comprising both $L_{2,1}$ normalization and a discriminant analysis is considered here [59]. Once the features are selected, it is incorporated with the concept of a majority voting scheme [60] and the features are selected before proceeding to classification. The overall illustration of the HFS-MVS classification model is shown in Figure 4 as follows.

#### 4.1.2. Hybrid SSO-PSO Algorithm for Feature Selection

To manage the feature vectors efficiently, a binary encoding is utilized by the feature set. A good and optimum feature set could be easily obtained by swarm optimization techniques so that the overall computation time could be less. The constructive search abilities of these algorithms are high when dealing in real time. Due to its robustness and versatility, PSO and SSO are selected in this work. In the entire search space, a solution is provided by the PSO depending on the particle and velocity. A global optimum result is provided by the PSO as the best positions are searched continuously by the particles. The problem of premature convergence occurs in PSO; it thereby affects the stabilization between the abilities of exploitation and exploration. So, the local optima trap can be prevented by incorporating SSO with PSO so that even a high performance could be obtained. While choosing the feature set, the hybrid optimization algorithm plays a vital role. The performance in the search space is improved by hybridizing SSO and PSO and the quality approximation of the optimal solution is improved.

*Salp Swarm Optimization (SSO):*

Depending on the foraging attitude of salps, this population-based algorithm was developed [61]. A chain structure is formed and a leader initiates the process while the rest of the salp follows the leader quietly. In every search space, the salp position is represented as s. The position of the food particles is analyzed as f where the optimal solution is projected as s. The simplified expression of SSO is formulated as follows:

(37) $$s_{n}^{\prime }=\left\{\begin{array}{cc} f_{n}+r_{1}\left(\left(u_{n}-l_{n}\right)r_{2}+l_{n}\right) & r_{3}\geq 0\\ f_{n}-r_{1}\left(\left(u_{n}-l_{n}\right)r_{2}+l_{n}\right) & r_{3}<0 \end{array}\right.$$

where in the $n^{th}$ dimension, the position of the leader is represented as $s_{n}^{\prime }$. The $f_{n}$ indicates the position of food particles, $u_{n}$ represents the upper-bound salp position and $l_{n}$ represents the lower-bound salp position. Within an interval of [0,1], the random variables are specified by $r_{1},r_{2}$ and $r_{3}$. The parameter $r_{1}$ helps to maintain the exploration and exploitation capabilities of salp and is mathematically expressed as

(38) $$r_{1}=2e^{-{\left(\frac{4c}{M}\right)}^{2}}$$

where the current number of iterations are represented as c and maximum iterations are represented as M. In a particular search space dimension, depending on its starting speed, the position of the salps which are following the leader can be changed.

*Particle Swarm Optimization (PSO):*

PSO helps to mimic the intelligent behaviour of foraging of the bird flocks [62]. In the entire search space, a swarm population is present and it comprises multiple particles. Unless an optimal solution is procured, the process continues throughout. For a particle, the assessment of position and velocity is performed as follows:

(39) $$z_{i}^{t+1}=z_{i}^{t}+v_{i}^{t+1}$$

where $z_{i}^{t}$ represents the $i^{th}$ particle position and $v_{i}^{t}$ represent the velocity vectors.

The position and velocity are updated as follows:

(40) $$v_{i}^{t+1}=jv_{i}^{t}+\alpha_{1}r_{1}\left(S_{best}\;of\;SSA_{i}^{t}-z_{i}^{t}\right)+\alpha_{2}r_{2}\left(G_{best}\;of\;SSA_{i}^{t}-z_{i}^{t}\right)$$

The initial $S_{best}$ and $G_{best}$ of SSO is utilized to update the position using the above Equation (40). The swarm search ability can be improved by adopting such a methodology and there is less probability of occurrence of local optima. The updation strategy of PSO is improvised so that the higher positions of the salp population are also improvised. To affect the best positions of the particles, two constants are used here such as $\alpha_{1}$ and $\alpha_{2}$. The $r_{1}$ and $r_{2}$ are random numbers assigned in the range of [0,1]. The parameter j is the weight parameter assigned which assesses the present velocity depending on the iteration count. The exploration ability of SSO and exploitation ability of PSO is enhanced by the position update easily so that both solutions can be located easily. Many local solutions can be avoided because of this hybridization, and only the best set of features can be produced.

**Figure 5:**
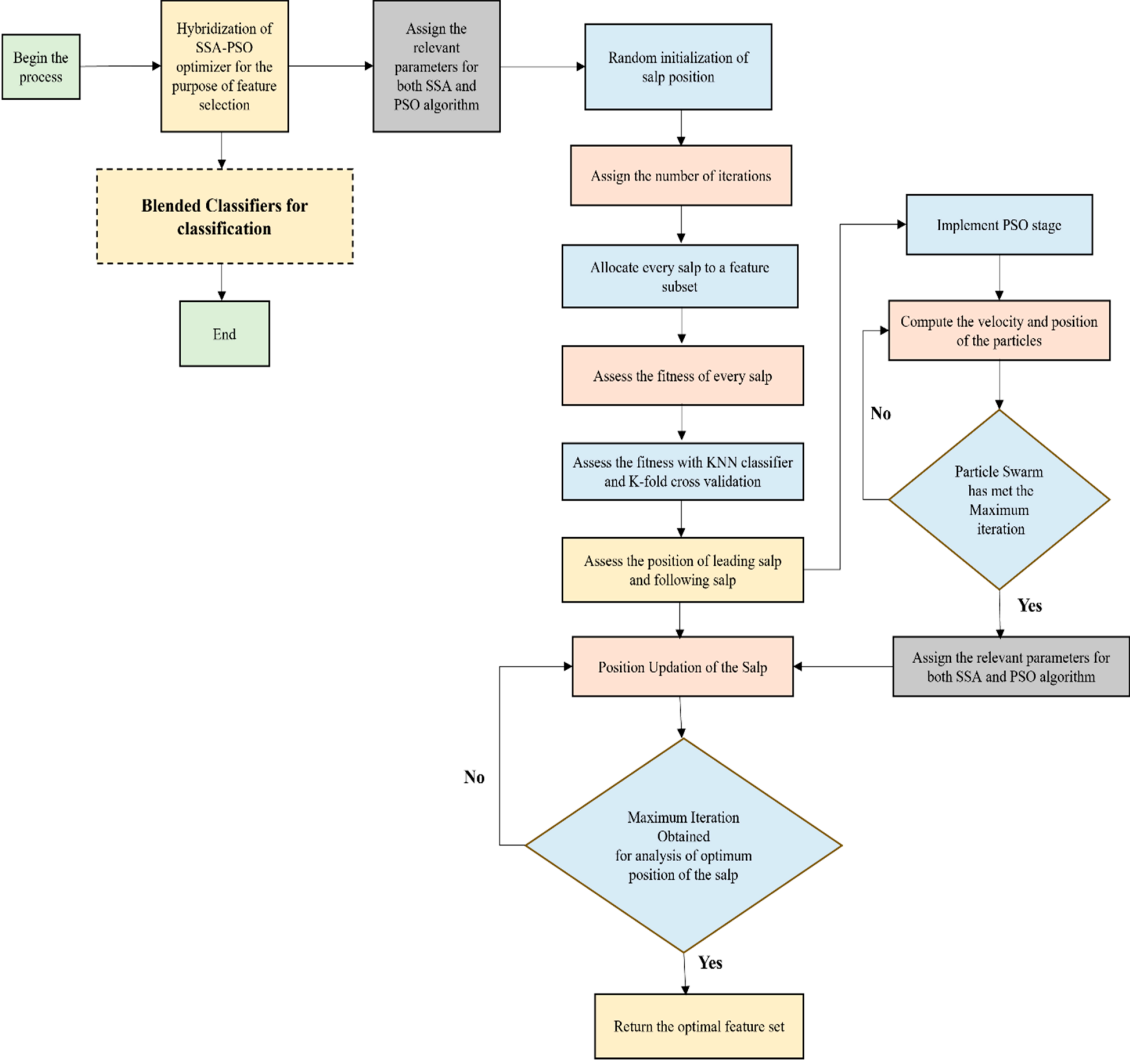
Simplified illustration of the hybrid SSO-PSO algorithm.

For the assessment of the hybrid optimizer, KNN output is used as an objective function. A solid balance must be maintained between the features and their respective performance. The fitness function is analyzed here as follows:

(41) $$Fitness=\gamma Err(D)+\beta \frac{\left|S_{i}\right|}{\left|F_{n}\right|}$$

where the significant features are denoted by $S_{i}$ and the total number of features are represented by $F_{n}$. γ and β are constants where the value is represented in the range of [0,1] that helps to assess the feature performance. The simplified illustration of the hybrid SSO-PSO algorithm is shown in Figure 5.

#### 4.1.3. Pearson Correlation Coefficient (PCC)

This technique is heavily dependent on the filter technique of the feature selection approach [63]. The range of PCC lies in the range of [−1, 1] and the association between the two continuous variables is quantified very well so that a stable relationship can be established well. Depending on the relationship between the target variable and the features, a good association is formed so that the input attributes are selected wisely.

#### 4.1.4. Mutual Information (MI)

This technique is also a type of filter method of feature selection approach where a score is provided for every feature [64]. Statistical analysis and measurements are used heavily here and then the attributes are formed initially. Once the attributes are formed it is then ranked depending on the score values in a descending format. A threshold value is set and then the feature subset is selected accordingly. The optimal feature characteristics are chosen and this technique requires less computation time too.

### 4.2. Classifiers Used in Blended Model 2

The classifiers used in the blended model include the PLS with LDA, SVM and Kernel-based classification techniques, ensemble classification model with soft voting strategy, and XGBoost classifier.

#### 4.2.1. PLS with LDA, SVM and Kernel-Based Classification Models

(A) PLS—LDA classification model:

There are quite a lot of variants of PLS and the basic algorithm considered for analysis is PLS with orthogonal scores in the context of statistical learning [65]. The modelling of the data matrix $P_{\left(n,k\right)}$ is performed so that the class response $q_{\left(n,1\right)}$ can be analyzed well, and it is performed with the help of q=Pβ+ε, where the PLS vector parameters are indicated by $\beta_{\left(k,1\right)}$ and is residual in nature. By means of scaling $P_{0}=P-1{\bar{p}}^{\prime }$ and $q_{0}=q-1\bar{q}$, the algorithm is initiated. PLS is considered as an iterative method where the components are utilized to indicate the iterations. The computation of weights, scores, P and q loading along with the deflation components P and q are performed in every iteration. PLS is utilized as a dimension reduction technique and so, computing PLS scores $S_{nPH}$ where H<k leading to n>H. Therefore, the small sample-sized data can be managed efficiently by utilizing classification techniques over the score of PLS. For the PLS—LDA-based classification, the execution of this algorithm is performed for a total of H number of iterations, where h=1,2,…,H. The computation of scores, weights, P and y loading, etc., in every iteration can be performed as follows. The calculation of the loading weights $w_{k\times 1}$ is performed as follows:

(42) $$w_{h}=P_{k-1}^{\prime }q_{h-1}$$

The covariance of $q_{h-1}$ is reflected by $P_{h-1}$. The loading weights are normalized so that the length is made equal to one by utilizing the following equation as follows:

(43) $$w_{h}\leftarrow w_{h}/\left\|w_{h}\right\|$$

The significance of sample-wise score $t_{n\times 1}$ is expressed as follows:

(44) $$t_{h}=P_{h-1}w_{h}$$

The data matrix is regressed by using the P loading indicated by $k_{k\times 1}$ in the $P_{h-1}$ on the score vector and indicated as follows:

(45) $$k_{h}=P_{h-1}^{\prime }\frac{t_{h}}{t_{h}^{\prime }t_{h}}$$

The Q-loading $v_{1\times 1}$ is represented as follows:

(46) $$v_{h}=q_{h-1}^{\prime }\frac{t_{h}}{t_{h}^{\prime }t_{h}}$$

The fitting of LDA is performed on the PLS scores S for the sake of discrimination, where $S_{c}$ scores are assumed for the class c and it has a normal distribution with a covariance Σ and mean $\mu_{c}$. The discriminant function is indicated as follows:

(47) $$\delta_{c}(S)=-\frac{1}{2}\log\left|\Sigma \right|-\frac{1}{2}{\left(S-\mu_{c}\right)}^{\prime }\Sigma^{-1}\left(S-\mu_{c}\right)+\log\pi_{c}$$

which leads to obtaining $q_{c}$.

The computation of the deflated components is specified as $P_{h-1}$ and $q_{h-1}$ is indicated as follows:

(48) $$P_{h}=P_{h-1}-s_{h}k_{h}^{\prime }$$

(49) $$q_{h}=q_{h-1}-s_{h}v_{h}$$

If h<H, the procedure is returned to the starting step itself. From every PLS iteration, the loading weights and scores are obtained and it is assimilated as follows:

(50) $$W=\left[w_{1},w_{2},\ldots ,w_{H}\right]$$

(51) $$S=\left[s_{1},s_{2},\ldots ,s_{H}\right]$$

(52) $$K=\left[k_{1},k_{2},\ldots ,k_{H}\right]\;\;\mathrm{and}$$

(53) $$v=\left[v_{1},v_{2},\ldots ,v_{H}\right]$$

In regression coefficient matrix, the results are presented as follows:

(54) $$\hat{\beta }=W{\left(K^{\prime }W\right)}^{-1}v$$

(B) PLS-SVM model:

A multivariate normal distribution must be followed by means of loading scores for class c $\left(S_{c}\right)$. Here, for every class, a common structure of variance-covariance is followed. The assumption of LDA is tough to follow if the sample size is very small and so, SVM is utilized as an alternative of LDA where a common structure of variance-covariance is not needed and the multivariate normal distribution scores $S_{c}$ are only required. Over the PLS scores S, an SVM-dependent classification is introduced so that high-dimensional data is classified with an exceedingly small sample size. For the sake of classification over PLS scores S, a hyperplane is built in an extremely high-dimensional space and is represented as follows:

(55) $$\vec{w}\cdot \vec{s}-h=0$$

where the normal vector to the hyperplane is represented by $\vec{w}$. The hyperplane offset present along the normal vector is assessed by the parameter $\frac{h}{\left\|\vec{w}\right\|}$. Kernel choices are important to assess the different SVM variants so that the PLS scores could be applied successfully over SVM algorithm.

(C) Kernel-based PLS-SVM model:

To improve classification accuracy, kernel tricks are utilized. If there is data separability issue, then the dimensions can be added so that the classification accuracy is improved. Various Kernels are present in SVM such as hyperbolic, Gaussian, Laplacian, Polynomial, Bessel, Linear, Radial Basis Function (RBF), Spline, etc. [66]. In this work, polynomial, Laplacian, Bessel, linear, and Spline Kernel-based PLS-SVM models are used.

The Polynomial Kernel-based PLS-SVM can be mathematically expressed as follows:

(56) $$k\left(s_{i},s_{j}\right)={\left(s_{i}\cdot s_{j}+1\right)}^{d}$$

The Bessel Kernel-based PLS-SVM can be mathematically expressed as follows:

(57) $$k\left(s_{i},s_{j}\right)=\frac{J_{w+1}\left(\left.\sigma \right|s_{i},s_{j}\right)}{{\left|s_{i}s_{j}\right|}^{-n\left(w+1\right)}}$$

The Laplacian Kernel-based PLS-SVM can be mathematically expressed as follows:

(58) $$k\left(s_{i},s_{j}\right)=e^{-\frac{\left|s_{i},s_{j}\right|}{\sigma }}$$

The Linear Kernel-based PLS-SVM can be mathematically expressed as follows:

(59) $$k\left(s_{i},s_{j}\right)=s_{i}.s_{j}$$

The Spline Kernel-based PLS-SVM can be mathematically expressed as follows:

(60) $$k\left(s_{i},s_{j}\right)=1+s_{i}\cdot s_{j}+s_{i}\cdot s_{j}\min\left(s_{i},s_{j}\right)-\frac{s_{i}+s_{j}}{2}\min{\left(s_{i},s_{j}\right)}^{2}+\frac{1}{3}\min{\left(s_{i},s_{j}\right)}^{3}$$

Therefore, five different Kernel-based PLS-SVMs are utilized in this work.

#### 4.2.2. Ensemble Classification Model with Soft Voting Strategy

To solve a particular issue, the understanding of multiple classifiers is required and it is obtained only by using an ensemble model. An accurate and good decision can be obtained by the ensemble model. Even if the performance of one classifier deviates, the performance of other classifiers can compensate for it, and so ensemble models have a higher classification power when compared to that of a single classifier. Four classifiers are used in this ensemble model, such as Decision Trees (DT), Naïve Bayesian (NB), Generalized Linear Model (GLM), and SVM.

(A) *DT:*

The user intervention is quite minimal when DT is used and so it is widely preferred in machine learning concept [67]. With the help of simple rules, the interpretation can be performed easily, and so it is quite famous for classification purposes. A root node initiates from a tree which helps to indicate a specific attribute. Branches are later developed based on the attribute value unless a particular value is obtained. Signal processing models developed on DT are quite versatile as multiple simple tests could be analyzed depending on a single prediction. The current outcome is mostly the consequence of previous outcomes at any levels in DT, and so a specific target could be achieved easily. For unobserved instances, the generalization is much better and a good understanding is provided by the tree architecture, proving its computational efficiency.

(B) *NB:*

Depending on Baye’s theorem, this simple and efficient classification model was developed [68]. An elevated level of scalability is provided by this classifier even when the feature space of input is much higher. For a clear analysis of the results, probabilistic knowledge is enabled by this classifier. The prior probability of a class is considered before any data is encountered by using the likelihood of the data. The mapping function can be assessed accurately so that a label of an unknown example can be predicted well during the classification analysis.

(C) *GLM:*

One of the famous parametric modelling schemes is GLM as probability interval can be predicted well by means of assuming the data distribution [69]. This model produces a variety of statistical indicators so that it is quite convenient for analysis and interpretation. A binary logistic regression model which has a logit link function along with a binomial variance has been utilized in this work. In between the dependent and obtained responses, the logistic regression classification model has identified a solid relationship. The predictors here mostly denote the explanatory variables and, with the help of the following equation, the logistic regression can be represented as follows:

(61) $$\mathrm{Logit}(\mathrm{Pr})=\log\left[\frac{\mathrm{Pr}}{1-\mathrm{Pr}}\right]=\alpha +\beta_{1}P_{1}+\beta_{2}P_{2}+\ldots +\beta_{n}P_{n}$$

The probability of outcomes is indicated by Pr. The regression coefficients are specified by $\beta_{1},\ldots ,\beta_{n}$. The descriptive predictors are indicated by $P_{1},\ldots ,P_{n}$ in this linear combination and the intercept value is denoted by α.

(D) *SVM:*

In this classification model, an optimal hyperplane can be easily found so that the margin can be maximized between itself and the corresponding training samples embedded in the high-dimensional space. The overall generalization error can be mitigated well in this classification model. No amount of information is required with respect to the correlation among variables during the prediction or classification process. The upgrading of the original model can be performed for the non-linear data by means of mapping the data into a higher dimensional space so that a hyperplane can be produced and it can separate the two different classes in that space. For classification of both small-sized samples and exceptionally enormous-sized samples, SVM is utilized and it can be suited well for the signal processing applications [52]. The training of the classifiers was performed individually, and then it was hybridized into various combinations depending on the soft computing technique which was incorporated successfully on the classification outcomes. The probability of outcome can then be assessed and analyzed successfully.

(E) *Soft Voting Computing:*

When classifiers have probabilistic outcomes, then they can be computed well with soft voting technique [70]. A weighted soft voting technique can be measured at an instance level or classifier level. The certainty of every classifier is considered carefully by the soft voting technique as more weights are assigned to this confident vote, and so soft voting is preferred often when compared to hard voting. The output probabilities are hybrid for every classifier, and then a final decision is computed. For every target class, the average probability is analyzed and the decision can be made. With the help of soft voting technique, the classifiers are hybrid and it is presented in Pseudocode 1 as follows:
**Pseudocode 1**: Soft Voting Computing and hybridizing classifiersInput: Features,
p-number of hybrid algorithms Output: Predicted class output Step 1: Start P_positive(i) indicates outcome probability is positive P_negative(i) indicates outcome probability is negative Step 2: for each iteration
i do For
n=1 to
p do If predicted class
(n) is positive P_positive(i) = P_positive(i) + probability
$\left(n\right)$ else P_negative(i) = P_negative(i) + probability
$\left(n\right)$ end if end for if P_positive(i) > P_negative(i) do Output predicted class = positive else Output predicted class = negative end if end for

For this ensemble classification model, the results can be easily analyzed and shown in terms of classification accuracy.

#### 4.2.3. XGBoost

If the optimized gradient boosting algorithm is improved, then it indicates an XGBoost algorithm which has a high probability, versatility, and efficiency [71]. It can be applied equally to both regression and classification tasks as it is a tree-dependent supervised algorithm. The conventional GBM framework is enhanced by the XGBoost algorithm by using multiple enhancements in the algorithm and optimization level. A parallelized tree-construction procedure is employed so that the sequential construction of trees is performed while equal focus is given on the concept of parallel computation. The concentration here is on a tree pruning method where trees grow to a maximum depth initially and, depending on a threshold of a loss function, it can be pruned back easily. Cache awareness concept is utilized by XGBoost so that an effective handling of both memory capacity and computational time is performed successfully. To avoid overfitting, the integration of regularization techniques is performed so that the model is regulated by means of reducing the coefficients. The missing values are handled quite efficiently and there is a smaller number of iterations required in this algorithm as it has an efficient cross-validation mechanism scheme. It is highly flexible, yet it requires extensive hunting. The model is enhanced and generalized by incorporating the concept of regularization concepts $\left(L_{1}\right)$ and $\left(L_{2}\right)$. The training process is faster when compared to GBM and the parallelization occurs clusters is made simpler with the help of this classification model.

## 5. Results

The experiment was conducted on a publicly available dataset obtained from 36 patients suffering from chronic neuropathic pain [30,31]. The patient pool comprised 28 females and 8 males, and the average age of the participants in this dataset was about 44 with a standard deviation value of ±13.98. Their pain levels were evaluated by means of completing two primary questionnaires. The two important conditions were present such as Pain detect Questionnaire (PDQ) and Brief Pain Inventory (BPI). The PDQ is a tool used for Spanish language validation and the neuropathic components of pain are used as well. The BPI is also a tool used for Spanish language validation and its primary form is on pain severity and its influence in the everyday life of the patient. Under three categories, the severity of pain can be stratified depending on the BPI scores such as low pain, moderate pain, and high pain. Using the standard 10/20 International system, the recordings of the EEG were conducted utilizing an EASYCAP electrode cap. With the aid of SMARTING EEG amplifier, the recordings are analyzed at a resolution of 24 bits, sampling frequency of 250 Hz, and a bandwidth ranging between 0.1 and 100 Hz. With the help of Openvibe software, signal acquisition was conducted. To minimize artefacts and noise and to ensure good data quality, an overall impedance level was assessed below 5 kΩ. The offline reference electrodes used here are the right $\left(M_{2}\right)$ mastoid and left $\left(M_{1}\right)$ mastoid. In a resting state, the recording was performed for both the conditions of eyes-open and eyes-closed cases. To maintain good data quality, patients with other neurological disorders were prevented from entering the investigation. In the dataset, it is made sure that the patients were given quality pharmacological treatments so that variability could be reduced, which is caused due to different medication effects.

A computer with 64 GB main memory, Intel i7 processor, 4.6 GHz processing unit, and MATLAB R2022b programming tool was utilized to perform the experiment. In our work, classification accuracy was analyzed in detail. A 10-fold cross-validation method was utilized in this work to compute the performance metric. As far as the parameters of the GWO are concerned, the population size is set to 200 and the number of iterations is set as 500 in our experiment. The population size is set to 250 in SMO algorithm; the global leader limit is assigned as 50 and the local leader limit is assigned as 75. The perturbation rate is fixed at 0.5 and the number of iterations is set as 500 in our experiment. When GBM is considered, the number of estimators is set as 100, the learning rate is set as 0.2, the number of subsamples is set as 1, max_depth is set as none, and the min_samples_leaf is set as 1 in our experiment. For SVM, the regularization problem is set as 1 and the kernels used in this work include linear, polynomial, RBF, sigmoid, etc. For CatBoost classifier, the hyperparameters are set as follows. The iterations are set as 500 and the learning rate is set as 0.08; the maximum depth of each tree is set as 4 and the L2 regularization term is set as 3. For PLS model, the number of components, also called latent variables, are set as 10 in this work. For SSO, the maximum number of iterations are set as 1000 as a higher iteration allows a good exploration and exploitation process. The population size is set to 600 in our experiment and the random coefficient are set in the range of [0,1] randomly. For PSO, an inertia weight of 0.5 is set and the cognitive constant value of 2 is set in the experiment. The social constant value is, again, set as 2 in our experiment. For XGBoost classifier, the eta value is set as 0.4, the gamma value is set as 0, and the max_depth value is set as 5 in our experiment. The number of samples are set as 1 initially and gradually increased, lambda value is set as 0.5 and alpha value is set as 0, and the max_leaves parameter are assigned as 4 in the experiment.

On examining Table 1, it is observed that for the DWT features, when selected with GWO feature selection technique and classified with SMO-GBM Classifier, a high classification accuracy of 92.18% is obtained. If Table 2 is analyzed, it is observed that for the DWT features, when selected with FCCT feature selection technique and classified with SMO-GBM Classifier, a high classification accuracy of 92.56% is obtained. On the analysis of Table 3, it is observed that for the DWT features, when selected with F-test feature selection technique and classified with SMO-GBM Classifier, a high classification accuracy of 89.45% is obtained. If Table 4 is analyzed, it is observed that for the DWT features, when selected with BOA feature selection technique and classified with SMO-GBM Classifier, a high classification accuracy of 87.34% is obtained. On examining Table 5, it is observed that for the FCM features, when selected with HFS-MVS feature selection technique and classified with Polynomial Kernel-based PLS-SVM Classifier, a high classification accuracy of 92.47% is obtained. If Table 6 is analyzed, it is observed that for the FCM features, when selected with SSO-PSO feature selection technique and classified with Polynomial Kernel-based PLS-SVM Classifier, a high classification accuracy of 92.68% is obtained. On the analysis of Table 7, it is observed that for the FCM features, when selected with PCC feature selection technique and classified with Polynomial Kernel-based PLS-SVM Classifier, a high classification accuracy of 86.11% is obtained. If Table 8 is analyzed, it is observed that for the FCM features, when selected with MI feature selection technique and classified with Polynomial Kernel-based PLS-SVM Classifier, a high classification accuracy of 85.68% is obtained. On examining Table 9, it is observed that for the DWT features, when selected with HFS-MVS feature selection technique and classified with ensemble classifier with soft voting method, a high classification accuracy of 91.56% is obtained. If Table 10 is analyzed, it is observed that for the DWT features, when selected with SSO-PSO feature selection technique and classified with ensemble classifier with soft voting method, a high classification accuracy of 92.45% is obtained. On the analysis of Table 11, it is observed that for the FCM features, when selected with PCC feature selection technique and classified with ensemble classifier with soft voting method, a high classification accuracy of 89.34% is obtained. If Table 12 is analyzed, it is observed that for the DWT features, when selected with MI feature selection technique and classified with ensemble classifier with soft voting method, a high classification accuracy of 87.45% is obtained.

**Figure 6:**
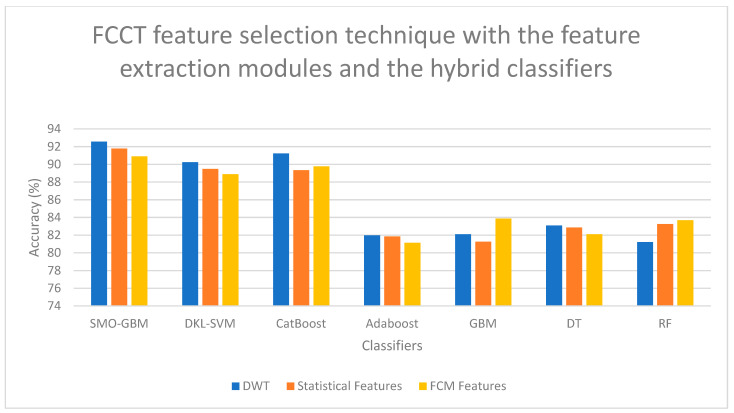
Performance Illustration (Accuracy) of the FCCT feature selection technique with the feature extraction modules and the hybrid classifiers.

**Figure 7:**
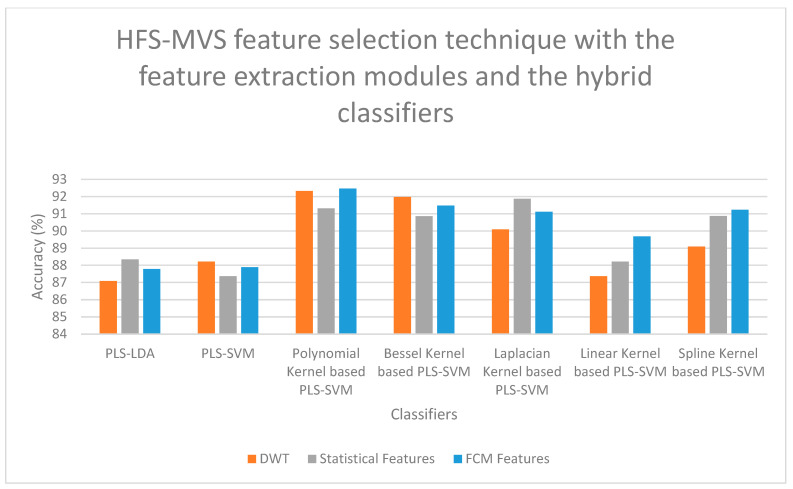
Performance Illustration (Accuracy) of the HFS-MVS feature selection technique with the feature extraction modules and the hybrid classifiers.

**Figure 8:**
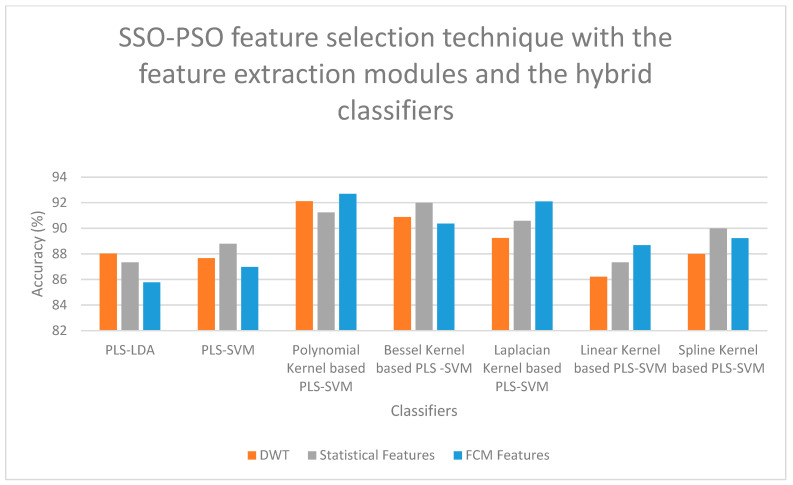
Performance Illustration (Accuracy) of the SSO-PSO feature selection technique with the feature extraction modules and the hybrid classifiers.

**Figure 9:**
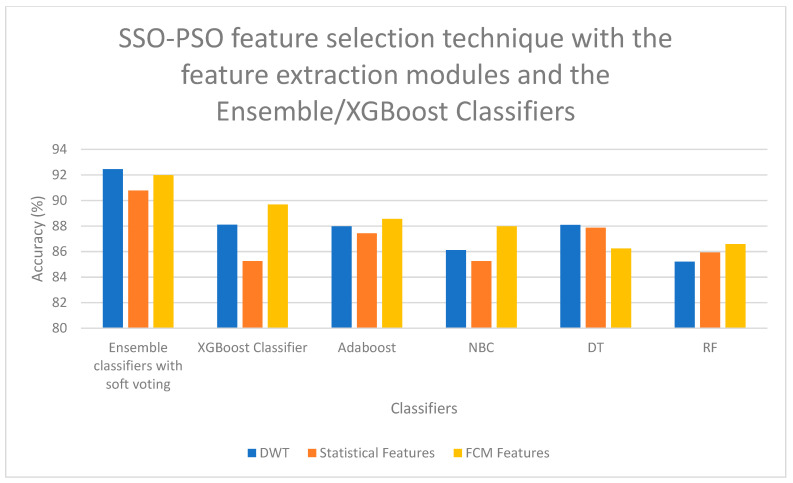
Performance Illustration (Accuracy) of the SSO-PSO feature selection technique with the feature extraction modules and the hybrid classifiers.

On examining Figure 6, a high classification accuracy of 92.56% is obtained for DWT features when selected with FCCT feature selection technique and classified with SMO-GBM Classifier, and a second-best classification accuracy of 91.78% is obtained for statistical features when selected with FCCT feature selection technique and classified with SMO-GBM Classifier. On examining Figure 7, it is observed that for the FCM features, when selected with HFS-MVS feature selection technique and classified with Polynomial Kernel-based PLS-SVM Classifier, a high classification accuracy of 92.47% is obtained, and a second-best classification accuracy of 92.33% is obtained for DWT features when selected for HFS-MVS feature selection technique and classified with Polynomial Kernel-based PLS-SVM Classifier. If Figure 8 is examined, it is observed that for the FCM features, when selected with SSO-PSO feature selection technique and classified with Polynomial Kernel-based PLS-SVM Classifier, a high classification accuracy of 92.68% is obtained, and a second-best classification accuracy of 92.11% is obtained for FCM features when selected for SSO-PSO feature selection technique and classified with Polynomial Kernel-based PLS-SVM Classifier. If Figure 9 is examined, it is observed that the DWT features when selected with SSO-PSO feature selection technique and classified with ensemble classifier with soft voting method, a high classification accuracy of 92.45% is obtained and the second-best classification accuracy of 91.98% is obtained when FCM features are selected with SSO-PSO feature selection technique and classified with ensemble classifier and soft voting methodology.

## 6. Discussion

The results of the present work are compared with the previous work and are tabulated in Table 13 for an elaborate understanding.

Not much work has been proposed in the literature for efficient classification of neuropathic pain from EEG signals. Out of the limited works proposed in the literature so far, a performance comparison has been made and reported in Table 13. On the analysis of Table 13, it is quite evident that the proposed works show good results when compared to the previous works. Though one work [37] has reported a very high classification accuracy of 99% and the current results do not match up with the results of that work, it is to be noted that, in this work, a variety of approaches have been blended efficiently and worked out together to check out its efficiency on the EEG signals for neuropathic pain detection and classification. The authors have tried hard to incorporate a variety of methods and models to see whether their hybridization can bring out some excellent results. Other than this work reported in [37], the results of the current work seem to have much improvement from the previously reported results for the three class classification scenarios. Most of the previously reported works have concentrated mostly on binary classification and the authors in this paper have tried hard to concentrate on three-class classification.

For the proposed blended models, the computational complexity was analyzed and it was found to be moderate only in this experiment in the range of $O(n^{3}\log n)$. As far as statistical tests are considered, the standard Cohen’s Kappa coefficient test and the 2-sided Wilcoxon test were conducted for the obtained features. All the feature values passed the good agreement category and ranged above 0.5 in this experiment when the Cohen’s Kappa coefficient test was implemented. A very high confidence level was attained for all the selected features when a 2-sided Wilcoxon test was implemented, showing the efficacy, suitability, and versatility of these features for classification. The computational time was computed and shown in Table 14 for the proposed models as follows.

On examination of Table 14, a least computational time of 4.213 s was obtained when FCM features are utilized with SSO-PSO feature selection technique and classified with Polynomial Kernel-based PLS-SVM Classifier. The next best computational time of 5.119 s was obtained when DWT features are utilized with FCCT feature selection technique and classified with SMO-GBM Classifier. The major limitation of this work can be said to be that this analysis was based on only on a small dataset, and in the future, the analysis and experimentation are planned to be conducted on massive neuropathic EEG datasets with deep learning analysis. The primary advantage of deep learning is that it aids in automatic feature learning and can handle large complex data with ease. The main drawback of deep learning is that it can be computationally intensive and it could perform poorly on any new data if it is not properly regularized. The hyperparameter tuning and managing the optimal settings like learning rate, layers, etc., can be challenging and iterative sometimes. Only when massive datasets are present, deep learning is applicable and for small datasets, deep learning is not suitable, and so, in the future, the implementation of this work will be applied to massive neuropathic EEG datasets with deep learning concepts.

## 7. Conclusions and Future Works

A patient’s life can be severely impacted with this chronic pain condition. The characterization of neuropathic pain has been reliant mostly on subjective perception, and so there are a lot of hinderances in the analysis of clinical decisions. The neuroplasticity factor is quite unpredictable and highly dynamic in nature, and so the characterization of neuropathic pain is complex to understand. In the field of clinical neuroscience, neuropathic pain detection and classification is of utmost importance with the help of machine learning. To understand the neural dynamics associated with neuropathic pain, EEG is highly useful, and when it is coupled with feature extraction and machine learning, it can work wonders. In this work, two approaches are proposed in terms of very efficient blended models for the classification of neuropathic pain through EEG signals. The best results are shown when the FCM features are selected with SSO-PSO feature selection technique and classified with Polynomial Kernel-based PLS-SVM Classifier, reporting a high classification accuracy of 92.68% in this work. The second-best classification result of 92.56% is obtained when DWT features are chosen with FCCT feature selection technique and classified with SMO-GBM Classifier. Future works aim to develop this model by incorporating a variety of other feature extractions, feature selections, and classification schemes. Also, deep learning is planned to be implemented by making a lot of modifications so that best results can be obtained. Future works also plan to implement this framework for telemedicine-based clinical applications.

## Figures and Tables

**Table 1:** Performance Analysis (Accuracy) of the GWO feature selection technique with the feature extraction modules and the hybrid classifiers.

	SMO-GBM	DKL-SVM	CatBoost	Adaboost	GBM	DT	RF
**DWT**	92.18	91.03	90.03	82.34	84.23	82.45	82.45
**Statistical** **Features**	90.04	90.56	89.45	80.48	82.09	81.11	83.26
**FCM Features**	89.34	89.35	88.46	80.45	82.31	83.02	82.47

**Table 2:** Performance Analysis (Accuracy) of the FCCT feature selection technique with the feature extraction modules and the hybrid classifiers.

	SMO-GBM	DKL-SVM	CatBoost	Adaboost	GBM	DT	RF
**DWT**	92.56	90.23	91.22	81.98	82.11	83.09	81.22
**Statistical** **Features**	91.78	89.47	89.34	81.85	81.26	82.87	83.25
**FCM Features**	90.90	88.89	89.76	81.13	83.87	82.11	83.68

**Table 3:** Performance Analysis (Accuracy) of the F-test feature selection technique with the feature extraction modules and the hybrid classifiers.

	SMO-GBM	DKL-SVM	CatBoost	Adaboost	GBM	DT	RF
**DWT**	89.45	87.67	88.45	83.09	80.21	82.09	80.11
**Statistical** **Features**	87.68	86.89	87.78	83.87	83.34	81.77	82.25
**FCM Features**	88.92	87.22	89.92	80.11	82.68	80.89	80.78

**Table 4:** Performance Analysis (Accuracy) of the BOA feature selection technique with the feature extraction modules and the hybrid classifiers.

	SMO-GBM	DKL-SVM	CatBoost	Adaboost	GBM	DT	RF
**DWT**	87.34	85.09	87.23	82.35	79.08	80.11	78.66
**Statistical** **Features**	85.56	84.89	85.45	82.67	80.77	78.24	79.78
**FCM Features**	86.77	86.33	86.77	79.82	80.81	80.79	81.25

**Table 5:** Performance Analysis (Accuracy) of the HFS-MVS feature selection technique with the feature extraction modules and the hybrid classifiers.

	PLS-LDA	PLS-SVM	Polynomial Kernel-Based PLS-SVM	Bessel Kernel-Based PLS-SVM	Laplacian Kernel-Based PLS-SVM	Linear Kernel-Based PLS-SVM	Spline Kernel-Based PLS-SVM
**DWT**	87.09	88.21	92.33	91.98	90.09	87.36	89.09
**Statistical** **Features**	88.34	87.36	91.31	90.86	91.87	88.21	90.87
**FCM Features**	87.78	87.89	92.47	91.48	91.11	89.68	91.23

**Table 6:** Performance Analysis (Accuracy) of the SSO-PSO feature selection technique with the feature extraction modules and the hybrid classifiers.

	PLS-LDA	PLS-SVM	Polynomial Kernel-Based PLS-SVM	Bessel Kernel-Based PLS-SVM	Laplacian Kernel-Based PLS-SVM	Linear Kernel-Based PLS-SVM	Spline Kernel-Based PLS-SVM
**DWT**	88.03	87.66	92.11	90.88	89.23	86.22	87.99
**Statistical** **Features**	87.34	88.78	91.23	91.98	90.58	87.34	89.98
**FCM Features**	85.78	86.98	92.68	90.36	92.09	88.67	89.22

**Table 7:** Performance Analysis (Accuracy) of the PCC feature selection technique with the feature extraction modules and the hybrid classifiers.

	PLS-LDA	PLS-SVM	Polynomial Kernel-Based PLS-SVM	Bessel Kernel-Based PLS-SVM	Laplacian Kernel-Based PLS-SVM	Linear Kernel-Based PLS-SVM	Spline Kernel-Based PLS-SVM
**DWT**	82.67	82.09	84.27	85.68	83.89	82.89	84.09
**Statistical** **Features**	81.96	81.11	85.48	85.82	84.98	81.54	83.82
**FCM Features**	82.85	82.25	86.11	84.44	84.22	80.23	83.44

**Table 8:** Performance Analysis (Accuracy) of the MI feature selection technique with the feature extraction modules and the hybrid classifiers.

	PLS-LDA	PLS-SVM	Polynomial Kernel-Based PLS-SVM	Bessel Kernel-Based PLS-SVM	Laplacian Kernel-Based PLS-SVM	Linear Kernel-Based PLS-SVM	Spline Kernel-Based PLS-SVM
**DWT**	80.89	81.09	85.21	84.32	82.65	80.26	82.09
**Statistical** **Features**	80.66	82.83	84.35	83.45	84.72	80.83	81.24
**FCM Features**	80.79	83.51	85.68	85.65	85.03	79.54	80.68

**Table 9:** Performance Analysis (Accuracy) of the HFS-MVS feature selection technique with the feature extraction modules and the hybrid classifiers.

	Ensemble Classifiers with Soft Voting	XGBoost Classifier	Adaboost	NBC	DT	RF
**DWT**	91.56	87.36	85.09	84.21	86.09	83.12
**Statistical** **Features**	90.76	86.87	84.34	85.35	85.88	83.44
**FCM Features**	91.11	88.11	86.89	85.47	87.96	84.78

**Table 10:** Performance Analysis (Accuracy) of the SSO-PSO feature selection technique with the feature extraction modules and the hybrid classifiers.

	Ensemble Classifiers with SOFT Voting	XGBoost Classifier	Adaboost	NBC	DT	RF
**DWT**	92.45	88.11	87.98	86.11	88.09	85.21
**Statistical** **Features**	90.78	85.25	87.43	85.26	87.87	85.92
**FCM Features**	91.98	89.68	88.56	87.98	86.24	86.58

**Table 11:** Performance Analysis (Accuracy) of the PCC feature selection technique with the feature extraction modules and the hybrid classifiers.

	Ensemble Classifiers with Soft Voting	XGBoost Classifier	Adaboost	NBC	DT	RF
**DWT**	88.67	89.12	87.33	86.09	85.21	84.29
**Statistical** **Features**	87.88	87.36	85.46	84.98	85.22	84.34
**FCM Features**	89.34	88.78	86.78	85.33	84.65	85.79

**Table 12:** Performance Analysis (Accuracy) of the MI feature selection technique with the feature extraction modules and the hybrid classifiers.

	Ensemble Classifiers with Soft Voting	XGBoost Classifier	Adaboost	NBC	DT	RF
**DWT**	87.45	86.09	84.21	83.56	83.09	82.21
**Statistical** **Features**	86.67	85.98	83.23	80.78	81.88	83.22
**FCM Features**	85.89	85.34	84.44	82.74	82.71	80.57

**Table 13:** Study of current results with previous work.

Authors	Year	Analysis Performed	Classification Type	Subjects Used	Accuracy (%)
Wydenkeller et al. [32]	2009	Analysis of clinical measures for neuropathic pain in spinal cord injury	Binary	26	84%
Vuckovic et al. [33]	2018	Prediction of neuropathic pain in spinal cord injury based on EEG classifier	Binary	41	86%
Anderson et al. [34]	2021	Higuchi Fractal Analysis of EEG signals for predicting neuropathic pain	Binary	20	80%
Bobby et al. [35]	2023	Quantum chaos butterfly optimization-based SVM for neuropathic pain detection from EEG	3 classes	28	77.72%
Miller et al. [36]	2024	Machine learning algorithms to analyze the presence of chronic pain using EEG	Binary	186	79.6%
Tasci et al. [37]	2024	Black-white hole pattern technique	3 classes	36	99%
Senturk et al. [38]	2025	Deep Autoencoders Latent features and Hybrid Mamba classifier	6 classes	36	Over 99%
Adebisi et al. [39]	2025	Brain Functional network approach with machine learning	Binary	36	Over 97%
Proposed Works with the best obtained results	-	*FCM features when selected with SSO-PSO feature selection technique and classified with Polynomial Kernel-based PLS-SVM Classifier*	3 classes	36	92.68%
		*DWT features when selected with FCCT feature selection technique and classified with SMO-GBM Classifier*	3 classes	36	92.56%
		*FCM features when selected with HFS-MVS feature selection technique and classified with Polynomial Kernel-based PLS-SVM Classifier*	3 classes	36	92.47%
		*DWT features when selected with SSO-PSO feature selection technique and classified with ensemble classifier with soft voting method*	3 classes	36	92.45%
		*DWT features when selected with GWO feature selection technique and classified with SMO-GBM Classifier*	3 classes	36	92.18%

**Table 14:** Computational Time Analysis for the proposed works.

Year	Authors	Methods	Computational Time
2025	***Proposed works***	*FCM features when selected with SSO-PSO feature selection technique and classified with Polynomial Kernel-based PLS-SVM Classifier*	4.213 s
		*DWT features when selected with FCCT feature selection technique and classified with SMO-GBM Classifier*	5.119 s
		*FCM features when selected with HFS-MVS feature selection technique and classified with Polynomial Kernel-based PLS-SVM Classifier*	7.517 s
		*DWT features when selected with SSO-PSO feature selection technique and classified with ensemble classifier with soft voting method*	6.998 s
		*DWT features when selected with GWO feature selection technique and classified with SMO-GBM Classifier*	5.9113 s

## Data Availability

The used dataset can be downloaded using (Zolezzi et al. 2023a, 2023b) Please refer to references [30,31].
